# Spontaneous rate of clonal single nucleotide mutations in *Daphnia galeata*

**DOI:** 10.1371/journal.pone.0265632

**Published:** 2022-04-01

**Authors:** Markus Pfenninger, Halina Binde Doria, Jana Nickel, Anne Thielsch, Klaus Schwenk, Mathilde Cordellier

**Affiliations:** 1 Department Molecular Ecology, Senckenberg Biodiversity and Climate Research Centre, Frankfurt am Main, Germany; 2 Institute for Molecular and Organismic Evolution, Johannes Gutenberg University, Mainz, Germany; 3 LOEWE Centre for Translational Biodiversity Genomics, Senckenberg Biodiversity and Climate Research Centre, Frankfurt am Main, Germany; 4 Institut für Zoologie, Fakultät für Mathematik, Informatik und Naturwissenschaften, Universität Hamburg, Hamburg, Germany; 5 Institute for Environmental Sciences, Universität Koblenz-Landau, Landau, Germany; Georg-August-Universitat Gottingen, GERMANY

## Abstract

Mutations are the ultimate source of heritable variation and therefore the fuel for evolution, but direct estimates of mutation rates exist only for few species. We estimated the spontaneous single nucleotide mutation rate among clonal generations in the waterflea *Daphnia galeata* with a short-term mutation accumulation approach. Individuals from eighteen mutation accumulation lines over five generations were deep sequenced to count *de novo* mutations that were not present in a pool of F1 individuals, representing the parental genotype. We identified 12 new nucleotide mutations in 90 clonal generational passages. This resulted in an estimated single nucleotide mutation rate of 0.745 x 10^−9^ (95% c.f. 0.39 x 10^−9^–1.26 x 10^−9^), which is slightly lower than recent estimates for other *Daphnia* species. We discuss the implications for the population genetics of Cladocerans.

## 1. Introduction

Mutations are not only an important source of genetic variation, but rather its origin. Therefore, they are one of the fundamental parameters to better understand molecular evolution. The per generation rate at which spontaneous mutations occur, as well as their mutational spectrum, influence many important evolutionary parameters and processes, including estimations of effective population size and genetic diversity [1–3], equilibrium of genomic base composition [4] and divergence time [5]. The *de novo* mutation rate determines the possibility, potential [6] and speed of adaptation [7] to different environmental conditions.

However, direct estimates of the mutation rate exist only for few species because the logistical challenges for such estimations are numerous. Recently, a new approach was introduced that allows rate estimations with reasonable effort [8]. Essentially, the approach combines the advantages of mutation accumulation lines (e.g. a high number of mutations to score) with those of the trio approach (e.g. short experimental duration), while avoiding their respective draw-backs (e.g. excessive length of experiment, high sequencing effort, respectively). Further, this approach was recently used to assess mutagenicity of anthropogenic substances [9]. We, therefore, adjusted this method here to estimate the clonal single nucleotide mutation rate of the water flea *Daphnia galeata*.

Species of the genus *Daphnia* served since decades as model organisms in ecology, evolution and ecotoxicology [10–12]. *D*. *galeata* belongs to the *D*. *longispina* species complex which dominates the zooplankton of many freshwater lakes in the Holarctic [13]. The species, like most *Daphnia*, reproduce via cyclic parthenogenesis [14]. For most of the time, the species reproduces asexually with a generation time of a few days, while sexual reproduction usually takes only place when environmental conditions deteriorate, usually once or twice a year. The large majority of generational passages are therefore asexual and likely govern the overall rate of mutational change in these species. The species was now supplemented with a highly contiguous genome draft [15] and other genomic resources [16], which allowed the estimation of the clonal single nucleotide mutation rate.

## 2. Material and methods

### 2.1.Setting up short-term mutation accumulation lines

We used three clonal lineages of *D*. *galeata* (M5, J2 and LC3.6) to start 24 short-term mutation accumulation lines (MAL). These clonal lineages were hatched from resting eggs sampled in sediment cores from Müggelsee, Lake Constance (both Germany) and Jordan Reservoir (Czech Republic, [10]) and maintained in the laboratory since. Details on the laboratory conditions for the general maintenance of *Daphnia* clonal lineages was previously described [17]. In short, single *Daphnia* individuals were cultured in 50 ml artificial *Daphnia* medium [18] at 18 +/- 1°C and a light:dark cycle of 16:8 hours. *Daphnia* individuals were fed three times a week with *Acutodesmus obliquus* (1 mg C/ml) and medium was changed weekly.

**Fig 1:**
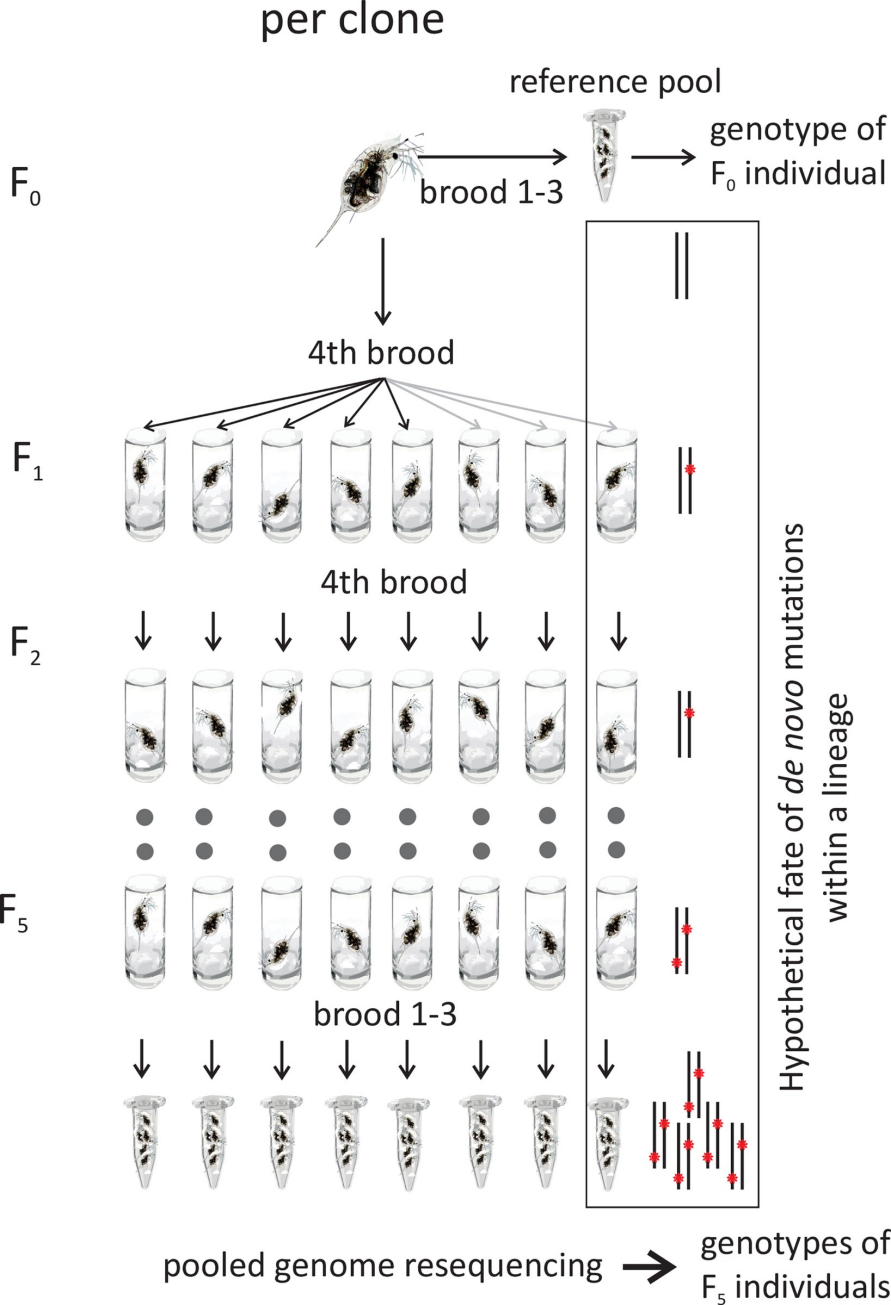
Schematic experimental set-up for the short-term mutation accumulation lines per clone. The fate of hypothetic *de novo* mutations for a single lineage is sketched in the box on the right.

A single individual from each of the three clonal lineages was chosen as F0 ancestor for eight replicate MALs each. As it is not possible to re-sequence the genome from a single individual, the produced broods 1–3 and 6–11 were raised, pooled and stored for sequencing. This followed the rationale that this ancestor reference pool represents the genotype of the ancestral individual, because mutations occurring in this first generational passage will not dominate the pool but rather appear in singleton reads. The MA-lines were then started from fourth and fifth broods, sisters to the F1 frozen for ancestor reference pool. This proceeding was maintained for the next four generational passages until generation F5. From this generation, all broods (up to sixteen, F6 individuals) were again pooled and used for re-sequencing. A schematic representation of the experimental design can be found in Fig 1.

### 2.2.Whole genome sequencing and bioinformatic processing

DNA was extracted for each pool of individual following a modified CTAB protocol, including a RNase step. The ancestor reference pool of each clone was sequenced to an expected mean coverage of 60X. After propagation for five generations broods of each of the MA-line was whole-genome sequenced to an expected mean coverage of 30X on an Illumina PE150 platform. Sequencing libraries were generated using NEBNext® DNA Library Prep Kit following manufacturer’s recommendations. The genomic DNA was randomly fragmented to a size of 350bp by shearing, then DNA fragments were end polished, A-tailed, and ligated with the NEBNext adapter for Illumina sequencing, and further PCR enriched with P5 and indexed P7 oligos. The PCR products were purified (AMPure XP system) and resulting libraries were analyzed for size distribution by Agilent 2100 Bioanalyzer and quantified using real-time PCR. Reads were individually adapter clipped and quality trimmed, using Trimmomatic [19]. Data was made available at ENA (acc. nos. ERS4993274-ERS4993294).

The cleaned reads of the ancestors and the MA lines were processed according to the GATK best practices guideline for pre-processing for variant discovery [20]. Reads were mapped with bwa mem [21] against the reference genome draft [15] and filtered using a combination of Picard tools v1.123 (https://broadinstitute.github.io/picard/) to mark the duplicates and GATK v.3.3.0 [20] for realignment around indels and recalibration of bases. The resulting bam files were then individually identified at the sample (SM:) field of the read-group tag.

The samples were then analyzed by two different software packages specifically designed to treat MA line data sets. AccuMUlate [22] makes use of a probabilistic approach to mutation calling that directly models the transition of alleles from the ancestral to descendants and accounts for heterozygous sites in all lines. It was previously validated in unicellular and complex metazoan animals as well as model and non-model organisms [9,22–25]. By its turn, muver [26] identifies mutation candidates with GATK’s HaplotypeCaller and applies several other processes to minimize false positive rates without losing sensitivity. It has the advantage of detecting indels but was validated only on *Saccharomyces cerevisiae* and with a human gold standard [26,27].

Prior to analysis with AccuMUlate, for each of the strains, ancestor and their respective MA lines were merged together with Picard’s MergeSamFiles generating three merged bam files: J2 (1 ancestor and 7 MA lines), M5 (1 ancestor and 7 MA lines) and LC3.6 (1 ancestor and 4 MA lines). With the reference genome and the following individual parameters for *D*. *galeata*: nucleotide frequencies of the reference genome (0.306 0.194, GC-content), probability of sequencing error (0.001, given a Q30 sequencing quality score), ploidy of descendants (2 = diploid) and ancestor (2), accuMUlate could then be run.

For muver, supporting files for the reference genome was prepared with the commands “index_reference” and “create_repeat_file”. Next, the bam files were processed together using GATK HaplotypeCaller tuned for high sensitivity (−-minPruning 0—minDanglingBranchLength 0 -A StrandAlleleCountsBySample). From this point on, the following commands were employed: “calculate-read-depths”, “calculate-bias-distribution” “calculate-depth-distribution”, “fit-repeat-indel-rates” and “call-mutations”.

Both softwares generate an output table where identified candidate mutations have a suite of statistics individually calculated. Those output tables are used to identify false-positive mutation calls. For accuMUlate it was filtered with a custom python script (full code is available in Supporting Information—S1 Appendix) according to the following rules: probability of a mutation/one mutation/of correct descendant genotype (> = 0.90); number of reads matching the putatively-mutant allele in samples that are not mutants (= 0); AD test statistic for mapping quality difference (< = 1.95); p-value from a Fisher’s exact test for Strand Bias and Pair-mapping rate difference (>0.05). The applied filtering step is considered conservative in comparison to previous studies using the same software [23] and is decisive to filter out false positive and inconclusive mutations associated with mapping error and low coverage areas. For muver, filtering consisted of accepting all non-NA values in the “SAMPLE Mutations” column and setting to 0 the “CONTROL_SAMPLE Genotyping Score”.

To assure sufficient coverage and minimize the effects of mismapped duplicated paralogous regions, individual coverage depth was restricted to the range of 15 to 45 reads. That way, total minimum depth ranged from 120x to 360x in the M5 and J2 lineages (min = number of MAL + ancestor 8 * 15X; max = number of MAL 8 * 45X) and from 75x to 225x for the LC3 lineage (min = number of MAL + ancestor 5 * 15X; max = number of MAL 5 * 45X. The individual bam files, together with the optimal individual coverage, were used to calculate the number of callable sites for each sample. The final candidate list from both outputs were then visually checked with the Integrated Genomics Viewer (IGV) [28] for final validation. This was done by verifying that the candidate mutation was not in complete association with alternate base calls in the candidate region, which is a characteristic of mismapped paralogs [29]. Candidates meeting this criterion were considered as false-positives and taken from the analysis.

To calculate the effective population size N_e_, we independently estimated Watterson’s theta (θ) [30] based on a sample that consisted of 12 resequenced *D*. *galeata* genomes from lake Dobersdorf [15]. We computed genotype likelihoods in ANGSD v0.931 [31] from BAM files aligned to the reference genome for all 4-fold degenerate sites using the SAMtools model (option–GL 1). Sites were filtered for a minimum mapping quality score of 30, a minimum base quality score of 20 and reads that had multiple mapping best hits or secondary hits were removed. The folded site frequency spectrum was calculated with the realSFS program and used as prior to estimate per-site Watterson’s θ for all sites using thetaStat implemented in the ANGSD package [31].

## 3. Results

From the 24 MALs, 18 produced enough offspring in the fifth generation to isolate sufficient DNA for re-sequencing. The MAL were sequenced to an overall mean coverage depth of 34.64 (s.d. = 4.47, minimum mean coverage = 22.45, maximum mean coverage = 42.86). Requiring at least four reads with an alternate allele to call a site heterozygous, truly heterozygous sites were therefore detected with a probability larger than 0.999. On average, 8.95 x 10^7^ sites (67% of the genome assembly, s.d. = 9.7 x 10^6^, min = 6.48 x 10^7^, max = 1.0 x 10^8^) were callable. In total, we scanned more than 1.6 billion diploid sites for mutations (Table 1).

**Table 1:** Information on the short-term mutation accumulation lines (MAL) from three clones of *D*. *galeata* investigated.

*D*. *galeata* clone	MAL	mean coverage	number of callable sites	number of mutations
J2	MA1a	35.25	95,267,650	0
	MA2b	36.85	88,640,972	1
	MA3a	30.37	72,474,255	0
	MA4a	41.88	64,808,013	0
	MA5b	42.86	80,960,726	0
	MA7a	35.27	95,314,644	0
	MA8a	31.86	89,598,402	1
LC3	MA2b	35.65	89,054,870	0
	MA3a	32.87	91,392,896	0
	MA6d	36.01	91,666,778	2
	MA7b	37.03	86,928,290	1
M5	MA1a	34.93	97,670,050	1
	MA2a	30.01	92,199,621	1
	MA3a	22.45	77,111,849	1
	MA5a	36.77	99,118,092	1
	MA6a	34.86	100,230,463	1
	MA7a	33.33	97,994,099	2
	MA8a	35.28	95,621,976	0
TOTAL	18		1,606,053,646	12

AccuMUlate had a better overall performance than muver in identifying single nucleotide candidate mutations. While accuMUlate identified 12 verified mutations, muver could identify only 6, being 4 of those overlapping with accuMUlate (Table 1 –Supporting Information—S2 Appendix) and 2 deletions of two and seven bases, on the clonal lineage M5 (MA7a > dgal17.201747 and MA8a > dgal76.247220). Both software solutions performed better when the above-described filtering options were applied (Table 2 –Supporting Information—S2 Appendix). The rate of false negatives after filtering was 16.67% for both methods. Meaning that one visually verified mutation in muver and two in accuMUlate were left outside the filtered list.

**Table 2:** List of single nucleotide mutation positions, their characteristics and effect.

*D*. *galeata* clone	MAL	scaffold	position	SNM	transition (ts) or transversion (tv)	amino acid change	gene function annotation
J2	MA8a	dgal52	163689	G > A	ts	-	-
	MA2b	dgal61	450819	C > A	tv	-	-
LC3	MA6d	dgal3	462655	G > T	tv	-	-
	MA7b	dgal98	307348	A > C	tv	-	-
	MA6d	dgal9	531976	C > T	ts	-	-
M5	MA5a	dgal9	390326	G > A	ts	P > L	Cellular nucleic acid-binding protein
	MA1a	dgal24	857256	A > C	tv	-	-
	MA7a	dgal40	527171	A > C	tv	-	-
	MA3a	dgal57	335627	C > G	tv	-	-
	MA7a	dgal57	589433	C > T	ts	-	-
	MA2a	dgal121	469817	A > C	tv	K > T	Density-regulated protein
	MA6a	dgal270	8936	G > T	tv	4 fold degenerate	unknown function

**Fig 2:**
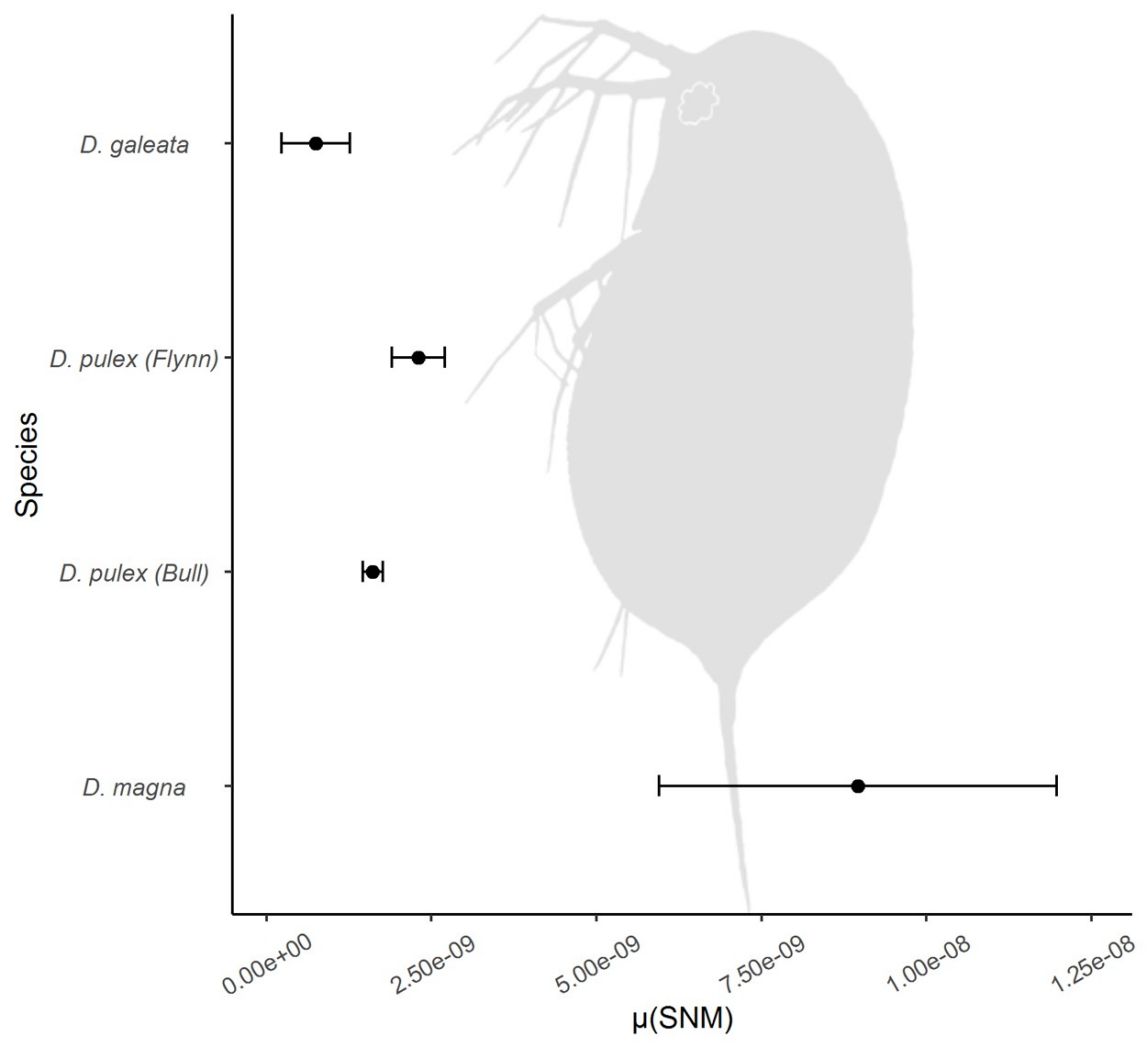
Haploid mutation rate (+/- 95% c.f.) of *Daphnia galeata* in comparison to other directly measured mutation rates of the genus.

Therefore, in the 18 MAL, we detected 12 single nucleotide mutations in 90 clonal generational passages (0.133 mutations per passage, Table 1). In line with expectations from the number of generations in the MAL, all observed *de novo* mutations occurred in heterozygous state. The rates among clones did not differ significantly (pairwise Poisson tests, p > 0.05 in all comparisons), therefore we report the single nucleotide mutation rate for all clones together. The haploid SNM rate μ was calculated as 0.745 x 10^−9^ (95% cf 0.39 x 10^−9^–1.26 x 10^−9^, Table 1). This rate was slightly lower than rates reported for *Daphnia pulex*, while all were substantially lower than the rate of *D*. *magna* (Fig 2). Using this rate, the mean θ estimate of 0.0092 and the relation θ = 4N_e_μ, the estimate for the long-term effective population size was 3.09 x 10^6^ (95% cf 1.83–5.90 x 10^6^) for *D*. *galeata*.

Three of the twelve observed mutations (25%) were found in exons of predicted genes (Table 2). This was within expectations given that the exon-space covers 22% and the gene-space 38.8% of the *Daphnia* genome assembly [15]. Of the three exon located mutations, one (dgal270.8936) was a synonymous G >C change at a 4fold degenerate site in a protein of unknown function. The two others resulted in non-synonymous changes. The G > A change at dgal9.390326 in a gene annotated as Cellular nucleic acid-binding protein caused an amino acid change from Proline > Leucine. A gene annotated as Density-regulated protein showed an A > C transversion (dgal121.469817) that resulted in a Lysine > Threonine exchange.

The ratio between A/T > G/C and G/C > A/T mutations was 7/4 = 1.75, which is in line with the observed GC content of 38.75% in the *D*. *galeata* genome. The ratio of transitions (4) to transversions (8) was 0.5, which is exactly the unbiased expectation.

## 4. Discussion

We report here for the first time a directly estimated clonal single nucleotide mutation rate for *Daphnia galeata*, a widely used model species. The obtained rate will significantly strengthen population- and comparative genomic approaches and serve as base line in evolutionary experiments of factors influencing the mutation rate. In contrast to other mutation rate estimates in *Daphnia* [32–35], which relied on MALs over several dozen generations, we have used the less time consuming short-term mutation accumulation approach recently devised [8]. While we obtained an accurate (low) single nucleotide mutation rate, the number of accumulated mutations was too low for meaningful analyses and comparisons of the mutational spectrum.

The spontaneous single nucleotide mutation rate of *D*. *galeata* reported here was slightly lower than rates estimated for *D*. *pulex* and much lower than for *D*. *magna*. It might be due to either the strict filtering steps applied or the short-term experiment, that unlike the classical approaches [33,34], gives less time for the lines to accumulate mutations that could, ultimately, promote the onset of other mutations. Therefore, our study is likely to be an underestimation of the *D*. *galeata* mutation rate.

The effective population size of the species was also the highest among the three species for *D*. *galeata* (N_e_ = 7.8 x 10^5^ in *D*. *pulex*, [36]and 4.2 x 10^5^ in *D*. *magna*, [34]). We found only few single nucleotide mutations per clonal generational passage (0.133), indicating a remarkable replication fidelity at first sight. One possible explanation could be high rates of loss-of-heterozygosity mutations [32], which would effectively remove half of the *de novo* mutations, while making the other half homozygous. But this process likely played no quantitative role here. Since ameiotic crossovers are exceedingly rare or absent in short experimental periods [37], it also leads to a neglectable number of insertion and deletion events or loss of heterozygosity during asexual reproduction in Daphnids [35,37]. Indeed, we did not observe homozygous *de novo* mutations and only two deletion events could be identified in just one clonal lineage.

However, we measured here the single nucleotide mutation rate per *clonal* generation. Given that *Daphnia* clones go through several clonal generations between sexual reproductions [14], the effective mutation rate is composed of the additive effect of all clonal generations between sexual generations. This cumulative mutation rate between sexual reproductions is therefore likely at least a magnitude higher as the clonal mutation rate (and the calculated N_e_ from this accordingly lower). This is the case even if the rate during sexual reproduction should differ from a clonal generational passage. Whether the use of a mutation rate estimate based on one parthenogenetic generation is appropriate to calculate the number of effectively sexually reproducing parents appears generally questionable. Previous study [36] found a 2–5 fold discrepancy between N_e_ and the efficiency of selection in *D*. *pulex* compared to *Drosophila melanogaster*, which may have its cause in using the single clonal mutation rate instead of the cumulative mutation rate between sexual reproductions.

Even though the number of single nucleotide mutations per clonal reproduction appeared to be low, this is put into a different perspective when considering the demography of the species. During peak densities, the number of individuals per square meter water column is in the order of 10^5^–10^6^ [38,39]. Even small lakes (say, 1 ha) therefore harbor billions of individuals (10^9^–10^10^). Assuming that the mutation rate inferred here also applies to natural conditions, a fraction of 0.133 of them carries a single nucleotide mutation relative to the previous generation. Therefore, the demographic peak generation in the hypothetical lake alone carries 1.33 x 10^8^–1.33 x 10^9^ newly arisen mutations. With an estimated total genome size of about 1.6 x 10^8^, each genome position is therefore hit mathematically between 0.8 and 8 times by a mutation in such a population. Even if the density may be lower in other lakes and varies within lake, it is reasonable to assume that evolution of populations at least in moderately sized lakes is not mutation limited. Every possible mutation is practically always present in the population and in larger lakes, perhaps even in every clonal lineage. This almost permanent presence of exhaustive genetic variation should offer excellent opportunities for adaptive tracking of changing environmental conditions [40], moreover since clonal reproduction should help to avoid stochastic loss of beneficial mutations [41,42]. Conversely, strong interclonal selection should ensure that genetic load remains low in the large natural populations of *D*. *galeata* [43]. In addition, the occasional seasonal sexual reproduction allows to recombine favorable variation together. This extraordinary, mutation-driven propensity of *Daphnia* for rapid adaptation may be the background for the observed monopolisation of lakes by particular clones [44].

## Supporting information

S1 Appendix Custom python script to filter the output from accuMUlate software. (DOCX)

S2 Appendix Comparison between accuMUlate and muver. (DOCX)
