# Supplementary material for: Spontaneous rate of clonal single nucleotide mutations in Daphnia galeata
Source: PLoS One. 2022 Apr 1;17(4):e0265632. doi: 10.1371/journal.pone.0265632 (PMC8975155; doi:10.1371/journal.pone.0265632)
Supplement: S1 Appendix — (DOCX) [file pone.0265632.s001.docx]

**Supporting Information**

**S1 Appendix -** **Custom python script to filter the output from accuMUlate software**

The code entails of sorting and pre-filtering the candidate mutations from the input .txt generated by accumulate.

It consists on:

'''

Created on May 11, 2020

@author: Markus Pfenninger

'''

#script to sort and filter candidate mutations

#import necessary modules

import re

import csv

from collections import Counter

#input files

infile = "….txt"

#necessary fields

scaffID = []

scaff_pos1 = []

scaff_pos2 = []

refbase = []

gen = []

mut = []

par6 = []

par7 = []

par8 = []

par9 = []

par10 = []

par11 = []

par12 = []

par13 = []

par14 = []

par15 = []

par16 = []

par17 = []

par18 = []

#Get infile, extract columns write them in a list, respectively

with open(infile) as to_read:

reader = csv.reader(to_read, delimiter = "\t")

for row in reader:

scaffID.append(row[0])

scaff_pos1.append(row[1])

scaff_pos2.append(row[2])

refbase.append(row[3])

gen.append(row[4])

mut.append(row[5])

par6.append(row[6])

par7.append(row[7])

par8.append(row[8])

par9.append(row[9])

par10.append(row[10])

par11.append(row[11])

par12.append(row[12])

par13.append(row[13])

par14.append(row[14])

par15.append(row[15])

par16.append(row[16])

par17.append(row[17])

par18.append(row[18])

print("read in infile")

outfname = "sorted" + str(infile) + ".out"

outf = open(outfname, "w")

#open outfiles

for i in range(0,len(scaffID)):

if int(par14[i]) == 0:

print("passed crit 1")

if float(par6[i]) >= 0.9:

print("passed crit 2")

if float(par7[i]) >= 0.9:

print("passed crit 3")

if float(par8[i]) >= 0.9:

print("passed crit 4")

if float(par15[i]) <= 1.95:

print("passed crit 5 candidate")

outf.write(str(scaffID[i]) +

"\t" + str(scaff_pos1[i]) +

"\t" + str(scaff_pos2[i]) +

"\t" + str(refbase[i]) +

"\t" + str(gen[i]) +

"\t" + str(mut[i]) +

"\t" + str(par6[i]) +

"\t" + str(par7[i]) +

"\t" + str(par8[i]) +

"\t" + str(par9[i]) +

"\t" + str(par10[i]) +

"\t" + str(par11[i]) +

"\t" + str(par12[i]) +

"\t" + str(par13[i]) +

"\t" + str(par14[i]) +

"\t" + str(par15[i]) +

"\t" + str(par16[i]) +

"\t" + str(par17[i]) +

"\t" + str(par18[i]) +

"\n")

outf.close()
