# Supplementary material for: Spontaneous rate of clonal single nucleotide mutations in Daphnia galeata
Source: PLoS One. 2022 Apr 1;17(4):e0265632. doi: 10.1371/journal.pone.0265632 (PMC8975155; doi:10.1371/journal.pone.0265632)
Supplement: S2 Appendix — (DOCX) [file pone.0265632.s002.docx]

**Supporting Information**

**S2 Appendix -** **Comparison between accuMUlate and muver**

Not all mutations identified by accuMUlate were called by muver. This probably happened because the GATK tools were designed and primarily serve to analyse human genetic datasets. Therefore, a pipeline based on the HaplotypeCaller tool are ultimately optimized for this purpose. Consequently, muver sensitivity on non-model organisms or with non-gold standard genome assemblies might vary greatly and be lower than expected. Further, because muver intentionally captures ambiguous mutation candidates to provide more information on copy number variation events, like large scale structural rearrangements or gene conversion (Burkholder *et al.*, 2018), low accuracy it is expected on determining point mutations and small indels.

Table 1 – Comparison between accuMUlate and muver on the called verified mutations

|  |  | AccuMUlate | muver | overlaping | |
| --- | --- | --- | --- | --- | --- |
| M5 | MA1a | 1 | 0 | NA |  |
|  | MA2a | 1 | 1 | 1 | dgal121:469818 |
|  | MA3a | 1 | 0 | NA |  |
|  | MA5a | 1 | 0 | NA |  |
|  | MA6a | 1 | 0 | NA |  |
|  | MA7a | 2 | 2 | 1 | dgal40:527172 |
|  | MA8a | 0 | 1 | NA |  |
| J2 | MA1a | 0 | 0 | NA |  |
|  | MA2b | 1 | 1 | 1 | dgal61:450819 |
|  | MA3a | 0 | 0 | NA |  |
|  | MA4a | 0 | 0 | NA |  |
|  | MA5b | 0 | 0 | NA |  |
|  | MA7a | 0 | 0 | NA |  |
|  | MA8a | 1 | 1 | 1 | dgal52:163689 |
| LC3 | MA2b | 0 | 0 | NA |  |
|  | MA3a | 0 | 0 | NA |  |
|  | MA6d | 2 | 0 | NA |  |
|  | MA7b | 1 | 0 | NA |  |
| total |  | 12 | 6 | 4 |  |

Table 2 – Accuracy of detection between AccuMUlate and muver

|  | accuracy | | | |
| --- | --- | --- | --- | --- |
|  | AccuMUlate | AccuMUlate + filtering | muver | muver +filtering |
| M5 | 63.63% | 100% | 36% | 80% |
| LC3 | 33.33% | 100% | NA | NA |
| J2 | 8.00% | 8.33% | 3.22% | 33.33% |

The accuracy was calculated considering the list of called candidate mutations and the final confirmation by IGV. Because no mutation could be confirmed on the LC3 line with muver, accuracy for it could not be calculated.
